# Simulating Defensive Trajectories in American Football for Predicting League Average Defensive Movements

**DOI:** 10.3389/fspor.2021.669845

**Published:** 2021-07-22

**Authors:** Marc Schmid, Patrick Blauberger, Martin Lames

**Affiliations:** Chair of Performance Analysis and Sports Informatics, Technical University Munich, Munich, Germany

**Keywords:** deep learning, Imitation learning, reinforcement learning, NFL analytics, data analytics, ghosting

## Abstract

American football is an appealing field of research for the use of information technology. While much effort is made to analyze the offensive team in recent years, reasoning about defensive behavior is an emergent topic. As defensive performance and positioning largely contribute to the overall success of the whole team, this study introduces a method to simulate defensive trajectories. The simulation is evaluated by comparing the movements in individual plays to a simulated league average behavior. A data-driven ghosting approach is proposed. Deep neural networks are trained with a multi-agent imitation learning approach, using the tracking data of players of a whole National Football League (NFL) regular season. To evaluate the quality of the predicted movements, a formation-based pass completion probability model is introduced. With the implementation of a learnable order invariant model, based on insights of molecular dynamical machine learning, the accuracy of the model is increased to 81%. The trained pass completion probability model is used to evaluate the ghosted trajectories and serves as a metric to compare the true trajectory to the ghosted ones. Additionally, the study evaluates the ghosting approach with respect to different optimization methods and dataset augmentation. It is shown that a multi-agent imitation learning approach trained with a dataset aggregation method outperforms baseline approaches on the dataset. This network and evaluation scheme presents a new method for teams, sports analysts, and sports scientists to evaluate defensive plays in American football and lays the foundation for more sophisticated data-driven simulation methods.

## 1. Introduction

American football is a widely used sport for the statistical evaluation of the performances of teams. Performance indicators for play-by-play data such as expected points added (EPA^1^), the defense-adjusted value over average (DVOA^2^), and defensive passing and rushing yards help to evaluate defensive plays (Cohea and Payton, 2011). Tracking data is also incorporated to evaluate single plays or specific game situations (Yurko et al., 2019). As demonstrated by recent Superbowl winners, defensive effectiveness has a major impact on winning. An emergent example for the acknowledgment of this fact is coach Paul Bryants' famous mantra:

Defense wins championships. Foxworth (2018)

Improving defensive behavior is, therefore, a major predictor for winning championships. Of the last eight Superbowl winners, four were ranked first or second in overall defensive rating in the league. In contrast to the offensive ratings, where just one team was ranked first or second. Hence, coaches' decisions on providing strategies for offense are important. However, defense is a key element for winning and statistics prove that^3^. It is cumbersome to imagine all possible defensive formations applicable for a specific offense. Furthermore, it is hard to determine which defender contributed to a specific defensive play, as defensive outcomes are commonly evaluated as a team achievement. With the emergence of tracking data, it is possible to cluster and classify specific contributions of defensive players, which helps to choose the right player in the corresponding play.

In 2013, the NBA team “Toronto Raptors” introduced a ghosting method to model the defensive behavior of opposing teams. These “ghosts” are synthesizing simulated trajectories of the movements of defensive players on the court. After 6 years of research, they developed a rule-based algorithm to simulate defensive behavior (Lowe, 2013). Unfortunately, this algorithm is not publicly available. This ghosting model computes more aggressive trajectories than observed during any NBA game, and only the most elite defenders (LeBron James in 2013 accounted for that) could mimic the behavior of the ghosts. Hence, the model seems unsuitable for imitating true defensive behavior. In recent years, research in artificial intelligence has leveraged methods to simulate human behavior by mimicking past motions and, therefore, better capture the movements of humans compared to a rule-based programming approach (Hussein et al., 2017). As offensive behavior implies interaction with highly unknown variables such as how the quarterback reacts (including creativity), passes or a scheduled game plan for the specific play, defensive behavior is mostly reactive and could, therefore, be modeled by imitation learning.

Modeling defensive behavior by simulating possible running trajectories of defensive players, knowing the behavior of the offense can be a notable tool. This could be used for setting up tactics beforehand or for the usage in retro-perspective analysis. Furthermore, this method can provide offensive and defensive coaches a tool to adapt their decision for the play strategy.

The presented ghosting model is capable of generating movement trajectories of the defensive teams *via* imitation learning, from the time the ball is snapped until the quarterback throws a pass forward. The model is evaluated using the expected pass completion probability at the moment the quarterback throws the pass forward.

The proposed model provides support for the decision-making of defensive coaches and helps with the evaluation of defensive strategies. It can be incorporated with media or fan applications or be used for extensive match analysis.

## 2. Related Work

The availability of tracking data in American football led to an increased amount of projects about evaluation and application engineering. The most commonly used tools in the area of team performance analytics are advanced statistical methods as well as machine learning and artificial intelligence. This chapter is divided into three parts, statistical methods, neural networks, and imitation learning.

*Statistical methods* have flourished in the past several years, and expanded the highly competitive landscape of sports analysis. Fernandez and Bornn (2018) modeled pitch control with a parametric approach to model influence areas of specific players with Gaussian functions and add the influence of each player to a team influence model based on the spatial coordinates on the field. Dutta et al. (2019) investigated defensive player behavior by classifying the behavior of defensive backs on two different coverage schemes, man coverage and zone coverage, using Gaussian mixture models to capture the state in an unsupervised manner.

Offensive player routes were analyzed with *neural networks* by recognizing and classifying running routes into different categories from wide out routes and backfield routes to compare the number of routes ran by the offense and the probability of targeting a receiver in that route (Team, 2019). Mehrasa et al. (2017) reduced player trajectories with one-dimensional convolutional neural networks for play recognition and team classification in basketball and ice hockey. The authors conclude, that franchise player or starting lineups contribute heavily to the team classification and identification using tracking data. Burke (2019) used deep neural networks to analyze the decision-making of quarterbacks and compute the pass completion probabilities of the quarterback with respect to the position of receivers and the closest defenders. Deshpande and Evans (2020) picked up this idea and extend the model in a more sophisticated way, by incorporating hypothetical pass probabilities in a Bayesian non parametric catch probability model. Most of the features of models regarding hypothetical passes are unobserved and, therefore, impute observable inputs.

*Imitation learning* yields multiple areas of operation in sport. Seidl et al. (2017) proposed a sketching tool for basketball play-by-play analysis, where they also use imitation learning to synthesize NBA defense. Coordinated multi-agent imitation learning was first proposed by Le et al. (2017) and was validated to be superior to an unstructured solution of a predator-prey problem, called the pursuit domain and on a soccer domain, where the results also showed a smaller loss in the coordinated case with respect to unstructured behavior. The training of the ghosted soccer players was done with Long Short Term Memory (LSTM) layers, while the Pursuit Domain was modeled with a random forest. A main finding of the study is the benefit of the alternating training of the model Hochreiter and Schmidhuber (1997) and the cascading training process of the LSTM layers for the problem.

The recently proposed methods and the extensive work by the NFL to make advanced statistics publicly available is the motivation to build a ghosting model for the defensive player trajectories of American football. Imitation learning is used to predict the trajectories of defensive players. Subsequently, these predicted positions are evaluated by comparison with the actual positions using a pass completion probability model.

## 3. Methods

In this study, a method to simulate individual and collective defensive behavior of American football players from the time of ball snap until the quarterback throws the pass forward is developed. A big aim of cornerbacks and safeties is trying to intercept passes or prevent offensive receivers from running the ball after the catch. Other defenders (e.g., linebacker, defensive end) try to rush and tackle the quarterback, so the pass cannot even be thrown. To date, to the best of our knowledge, no model accounts for the different team strategies or the contribution of individual defensive players to the outcome of the play.

The proposed ghosting model takes advantage of a comprehensive representation of tracking data. Individual defensive players are modeled with the positional information of the offensive team. As ghosted trajectories do not behave like the true running trajectory, a learned pass completion probability model, similar to previous study (Burke, 2019; Deshpande and Evans, 2020) is proposed to evaluate the true running trajectories with the synthesized trajectories.

### 3.1. Data

**Figure 1:**
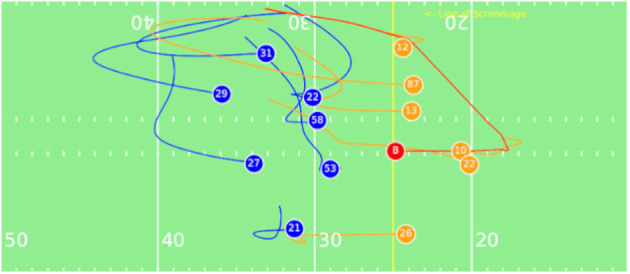
Motivational Play: Eli Manning (10) throws a pass short right to Cody Latimer (12) for 8 yards before stopped by a tackle. The the trajectories of offensive teams are displayed in orange, the trajectories of defensive teams are displayed in blue, and the ball trajectory in red. The line of scrimmage is labeled and displayed in yellow.

In December 2020, the NFL released a free-to-use, new NFL player and ball tracking dataset for the NFL Big Data Bowl 2021 challenge (NFL Big Data Bowl, 2020). The dataset includes game data of 17 weeks of the 2019 regular season of the NFL. Each game contains play-by-play positional information about defensive and offensive players and football, as well as meta-information about the play as illustrated in Figure 1. Positional information is provided for different numbers of players, ranging from 10 players to 21 players. These players are tracked by a radio-frequency-based system (RFID). The sensors were implemented in both of the shoulder pads of the player, to capture the position of the player as well as the upper body orientation at a rate of 10 Hz. Compared to current optical tracking systems used in basketball, hockey, and soccer, RFID-based tracking in American football is error resistant, and it is possible to measure accurate positions and orientation, even with visual indentations. The manufacturer states an accuracy of 6 inches (≈ 15.24 cm). However, to the best knowledge of authors, no validation study evaluating the accuracy is published yet of the system. The recording starts when the offense is set, meaning that the motion of offense and the reaction of the defense before the ball are snapped, are also captured in the data. For each tracked player and ball, every time frame contains its x and y position on the field within 0*m* ≤ *x* ≤ 120*m* and 0*m* ≤ *y* ≤ 53.3*m*. The speed and orientation of the upper body of each player are saved to individual vectors. Offensive players also have an attribute for the running routes (e.g., Go, Hitch, and Crossing). For every player, different time frames are marked with the respective events, i.e., when the ball is snapped, the quarterback throws a pass, the pass is received, or the first contact with the defender.

### 3.2. Pass Completion Model

**Figure 2:**
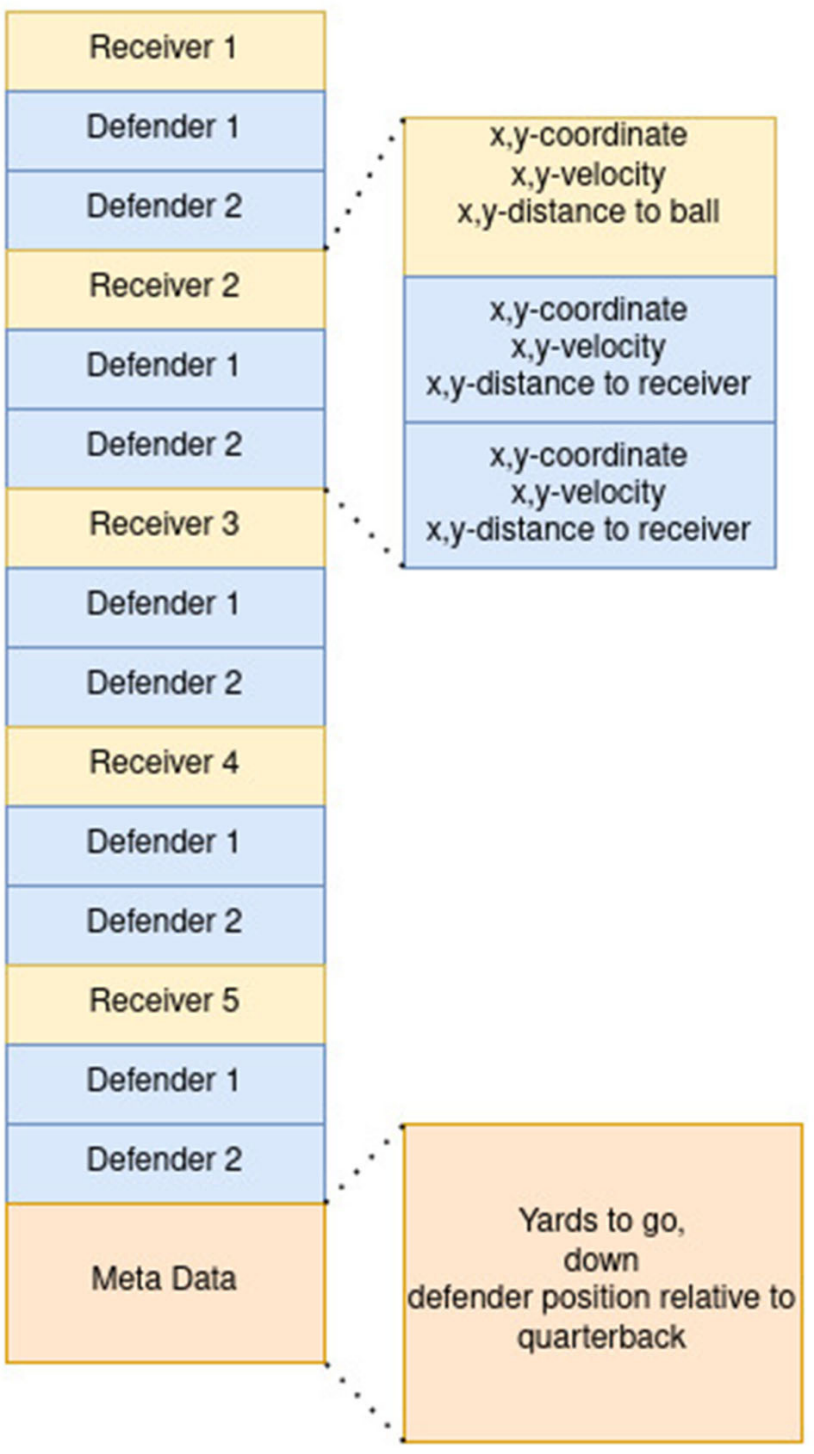
Feature Vector of the pass completion model.

Pass completion can be modeled in various ways. The NFL introduced a model to evaluate pass completion probabilities of specific players based on 10 features corresponding to every receiver (Team, 2018). With this method, it is difficult to simultaneously evaluate the positions all players', as every single route is computed and player-to-player comparison is conducted. Consequently, a single evaluation metric cannot be generated without engineered adjustments. To circumvent this issue, the pass is captured as a binary problem for the entire team in this study. This simplification helps to capture the completion probability and combines the probabilities of player-to-player single routes analysis in a model where the different routes are automatically combined in an end-to-end approach. In the model, *y* captures whether the pass was caught, given the specific formation and speed of the players, neglecting the targeted player. The following formulas illustrate that this issue can be considered a binary classification problem with a completion probability:

(1) $$\begin{array}{l} P\left(y=1|\text{X}\right)=\frac{1}{1+e^{-f\left(\text{X}\right)}} \end{array}$$

where **X** is the feature vector containing the positional information of all players and is defined according to Figure 2, and *f*(**X**) is to be optimized by a logistic regression

(2) $$\begin{array}{l} \log\left(\frac{P\left(y=1|\text{X}\right)}{1-P\left(y=1|\text{X}\right)}\right)=f\left(\text{X}\right) \end{array}$$

As a universal function approximator of *f*(**X**), feed-forward neural networks with different architectures are used, which are optimized by a grid search and are compared to a gradient boosted tree model, likewise with optimized hyperparameters by a grid search. Additionally, the problem can also be viewed as multi-class classification, yet as interception probability is low and is linked to large noise, as discussed by Burke (2019) and Deshpande and Evans (2020), it is possible to neglect the special classes in this application and further classify the pass outcome as positive or negative.

The neural network was trained with the ADAM optimizer (Kingma and Ba, 2014), while learning rate and architecture were selected with a grid search resulting in the architecture of three fully-connected layers with batch normalization. The first two layers were of size 64 with an additional dropout layer, while the last layer is a fully-connected layer with 32 neurons. The batch size was kept at 1,024 samples per batch and the learning rate was chosen to be 2e-3. The gradient boosted tree hyperparameter search for the architecture resulted in a maximum depth of 10 and 60 leaves. The learning rate is 0.03. The models were trained with 7-fold stratified cross-validation. Furthermore, a focal loss was used to account for class imbalances, yet, this did not yield better results and was not used/chosen following the law of Occam's razor.

#### 3.2.1. Feature Vector and Training Data

The training, validation, and test data consists of all passes with seven tracked defenders and six tracked offensive players, either complete or incomplete/intercepted from the NFL Season 2019. The training and validation data consists of the first 14 weeks of the NFL regular season, while the test data was taken from the last 3 weeks of the regular season. Overall, 17,346 passes were conducted. After filtering to the specific conditions, 9,199 passes were left. The whole training/test dataset was split 70/30% to accurately train the model and minimize overfitting.

The feature vector was created with respect to the available data from the later proposed ghosting model. As the ghosting model will synthesize the trajectories from the event of ball snap to the moment when the quarterback is throwing the ball, the latest possible time step to determine the pass receiving probability, the event of the forward pass, is used. Besides this information, each of the five receivers is assigned with the relative and absolute position of the two closest defenders as proposed by Burke (2019). The feature vector is ordered as shown in Figure 2. The quarterback was handled as a separate feature collection, as the distance to itself is irrelevant. Furthermore, a relative position on the field regarding the yard line and the down and yards until the next down starts was added.

The training set was augmented to make the receiver input order invariant by randomly changing the input receiver position in the feature vector. Furthermore, the play was normalized to always face in one direction and the line of scrimmage is the original orientation regarding the *x*-axis. This can be done, as the play itself should be rotation invariant, and the outcome should not depend on which direction the quarterback throws the pass.

### 3.3. Ghosting/Deep Imitation Learning

In Le et al. (2017) presented a ghosting model for soccer teams, learned from a season of professional soccer data *via* deep imitation learning. This model was able to capture team behavior in response to different attacking scenarios. In addition to useful insights for team comparisons, the trajectories also produced seemingly trivial outputs upon visual inspection in the first few seconds. However, it is precisely these few seconds in American football that provide insight into defensive behavior, which is why the deep imitation learning approach is transferred to American football and the focus is exactly on these first seconds, because they are essential to the defensive behavior before the pass takes place.

#### 3.3.1. Data and Feature Vector

In this part of the study, the NFL Next Gen Dataset of the 2019 Season is used and the games are filtered for plays with seven defensive and six offensive players and, therefore, 14 trajectories with the ball. Moreover, the vector contains meta-data regarding yards to go until the next down, number of downs, and absolute distance until touchdown. This results in the same number of training sequences as for the completion probability model, all plays are filtered, where there was no forward pass, the quarterback was not sacked and did not perform a handoff. The average sequence has a duration of 3.6 s.

**Figure 3:**
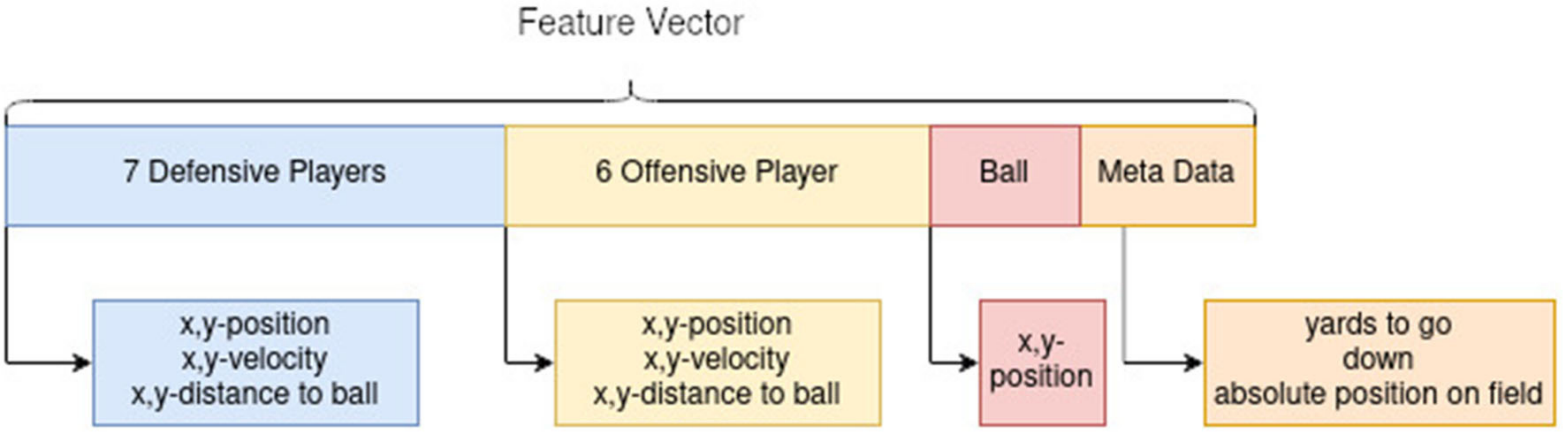
Feature vector ghosting model.

**Figure 4:**
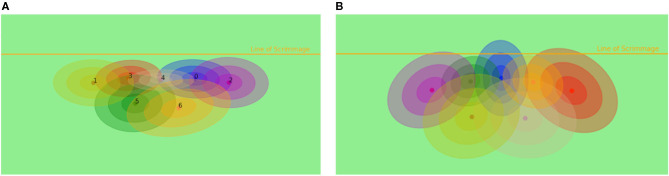
**(A)** Assignment of specific roles in formations with Gaussian mixture models to the corresponding feature vector. **(B)** Assignment of roles with a learned Hidden Markov model with Gaussian emission functions for different roles to the specific model according to running positions in the coordinated multi-agent approach.

The feature vector of the model is ordered first by the defensive team, then by the offensive team, followed by the ball position and the meta-data, which includes yards to go, number of downs and the absolute position on the field, where the play takes place. The position of players is again normalized so that the line of scrimmage determines the *x*-axis, and the play takes place in the negative y-direction from the perspective of the offensive team. This is displayed in Figure 3. As there is no formation information in the NFL NextGen dataset, a consistent representation of the position of players and positional behavior in the feature vector needs to be guaranteed. To achieve consistency, the tactical role of each player was assigned independent of their named position. Accordingly, an unsupervised role alignment algorithm (Gaussian mixture models) was chosen. The idea of inferring to a specific formation was developed and discussed by Bialkowski et al. (2016). The roles get assigned with a Hungarian algorithm, where a Gaussian mixture model is trained on starting positions and assigns the initial roles according to it. The model is illustrated in Figure 4A. This improves the structure of the learning problem, as defensive backs can switch positions and safeties can act as defensive ends. Moreover, cornerbacks and safeties are not bound to be on the left or right side of the field, so assigning positions in a fixed value might disrupt the network and make the learning problem impossible.

#### 3.3.2. Training With Imitation Learning

Imitation is the ability to recognize and reproduce others actions^4^. Hence, imitation learning is learning and developing new skills from observing these skills performed by another actor or oracle. The agents in multi-agent imitation learning contribute individually to a specific goal and need to collaborate.

This multi-agent imitation learning problem arises from two factors, first multiple agents need to learn simultaneously, and the role assignment of the learned agents dependent on the action of the corresponding model, which is in regard again dependant on the assigned role. To overcome this interdependence, Le et al. (2017) proposed an alternating optimization approach, by first optimizing for the imitation task, with a fixed role assignment, next fixing the policies and retrain the assignment model. This approach is repeated until no further improvement takes place on the validation set.

The assignment model, also called the structured model by Le et al. (2017), is learned *via* an estimation maximization algorithm on a Hidden Markov model with Gaussian emissions, and training was conducted on the same data as the other parts of the algorithm, despite velocity, distance to quarterback and ball position were not used to cluster the trajectories. The results are displayed in Figure 4B.

When learning variable-length sequences, recurrent neural networks are suited well for this job. LSTM layers are preferably used to model sequences of this kind and are eminently used when long-term dependencies are playing roles in current predictions. The individual trajectories are modeled with a two-layer LSTM with 128 neurons each. In training, the sequences were split into a length of 25 and an overlap of 10. Later, role-based model learning is compared to static model learning.

The models are trained in three phases according to Le et al. (2017): pretraining, single policy training, and joint policy training. When pretraining, the models are trained with a least-square learning approach without interaction of the single model itself or with other models. This means the model predicts the next timestep of the players, given perfect information and correction of the miss-predictions in training. This method does not resemble realistic trajectories, but initializes models parameters well for the following tasks. In the next step, the policies predict multiple timesteps, with imperfect information of their position, yet all other players have perfect information. This process of predicting multiple timesteps into the future is called rollout. The error of the imperfect information prediction is used to update the model again, and it helps to recover the model from ill predictions. This results in stable position predictions of the policy and enables the model to recover from prediction mistakes and eventually simulates test time during training first introduced by Ross et al. (2011) under the terms of no-regret online learning and present it under the term DAgger (Dataset Aggregation method). In the last step, all ghost models are trained together by predicting the respective next position on the field. Therefore, every model updates the corresponding training data input by imputing the predicted role positions and, therefore, simulating the complete defensive behavior. Empirically, this generates more stable trajectories. The joint training seems to make the model more robust against perturbations in general.

## 4. Results

### 4.1. Pass Completion Probability

The pass completion probability is used to validate the proposed ghosting model and, therefore, needs to be appropriately calibrated. As the baseline for the classification problem, a naive classifier of assigning every pass as a catch is used. The result of this method is comparable to the mean pass completion rate in the NFL for the test set data and accounts for 64.8%. First experiments of the pass completion probability model yield disillusioning results, when using ordered data as described by Lucey et al. (2014), as the highest accuracy of the best model is <5% better than the naive classifier.

**Figure 5:**
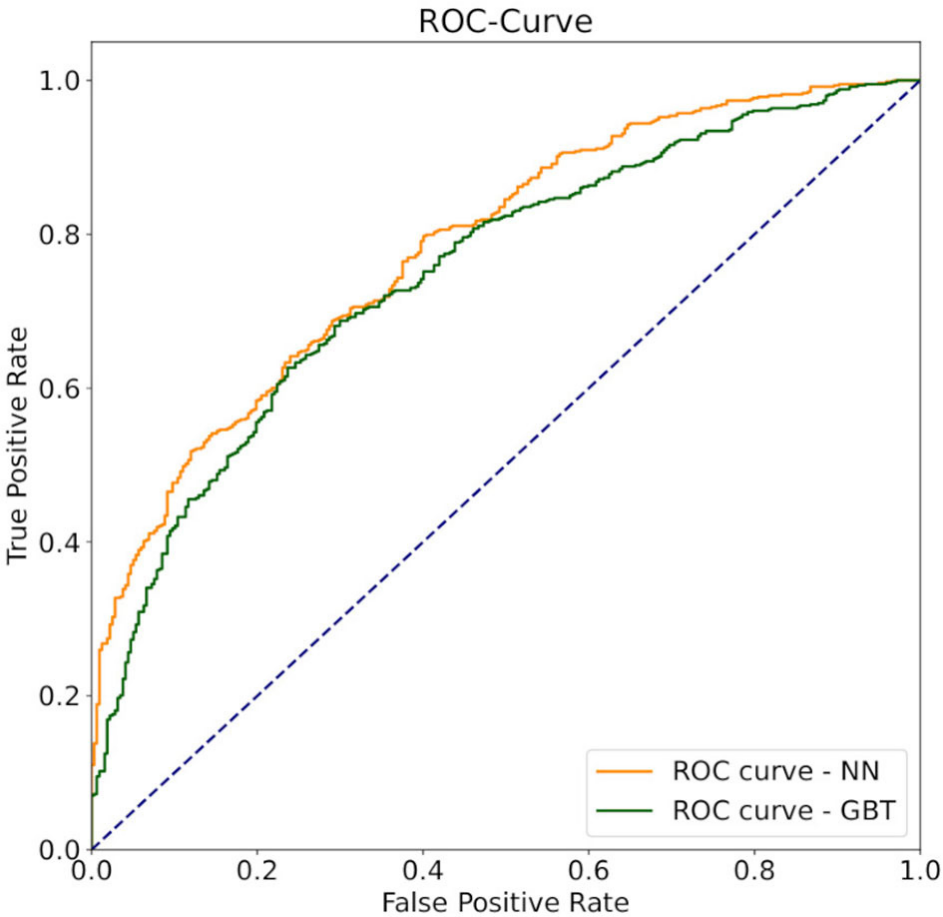
The ROC curve of the pass completion probability model. The AUROC is 0.746 for the neural network and 0.730 for the gradient boosted tree. The dashed blue line, represents the baseline of a random sample, the orange line, the ROC curve of the neural network, and the green line represents the ROC curve of the gradient boosted tree.

In other fields like quantum mechanical force prediction with black box estimators, order invariant learning is important. To achieve this, the atoms are either ordered by distance (Behler and Parrinello, 2007) or the invariance is learned by random permutations (Bapst et al., 2020). When applying random permutations to the order of the receivers, the accuracy of both models, neural networks and gradient boosted trees increases. The neural network outperforms the gradient boosted tree by around 5% in-accuracy (Table 1). In Figure 5, the Receiver operation characteristics (ROC) curve for the final classification models, with Area under ROC (AUROC) scores of 0.746 and 0.73 respectively, are displayed. The curve shows how well the signal is separated from the noise and returns another evaluation metric for binary classification problems. According to Hosmer and Lemeshow (2000), an acceptable value for the discrimination ability of binary classification is defined between 0.7 < AUROC < 0.8. Rice and Harris (2005) are arguing that AUROC > 0.714 can be classified as good and AUROC > 0.639 as acceptable. Hence, the used classification models are suitable for evaluating the ghosting model. For the following evaluation, the neural network approach was chosen due to the higher AUROC and accuracy score.

**Table 1:** Table comparing the accuracy of pass completion prediction and the correlating miss-classification rate.

**Model and data**	**Accuracy**	**Miss-classification**
Neural Network, ordered Data	69.5%	30.5%
Neural network, order-invariant data	81.6%	18.4%
Gradient boosted tree, order data	66.9%	33.1%
Gradient boosted tree, order-invariant data	76.2%	23.8%

### 4.2. Ghosting

For ghosting models to have value, they should conscientiously represent true behavior. In the first step, the exemplary results of the model are qualitatively evaluated, and examples of different behavior produced by the ghosting model are discussed. Finally, the models are checked in terms of prediction accuracy and precision. This means that the true (x,y) position of the players is compared with the predicted (x,y) position.

As discussed by Le et al. (2017), the task of the same player can vary throughout a play. By validating this hypothesis, a coordination model and a team-based model without coordination were trained. The coordination model alternately updates, also called cross-update, the chosen policy with the hidden Markov model, displayed in Figure 4B, while the team-based model assigns the feature vector, and hence the formation, with a Gaussian mixture model, displayed in Figure 4A.

**Figure 6:**
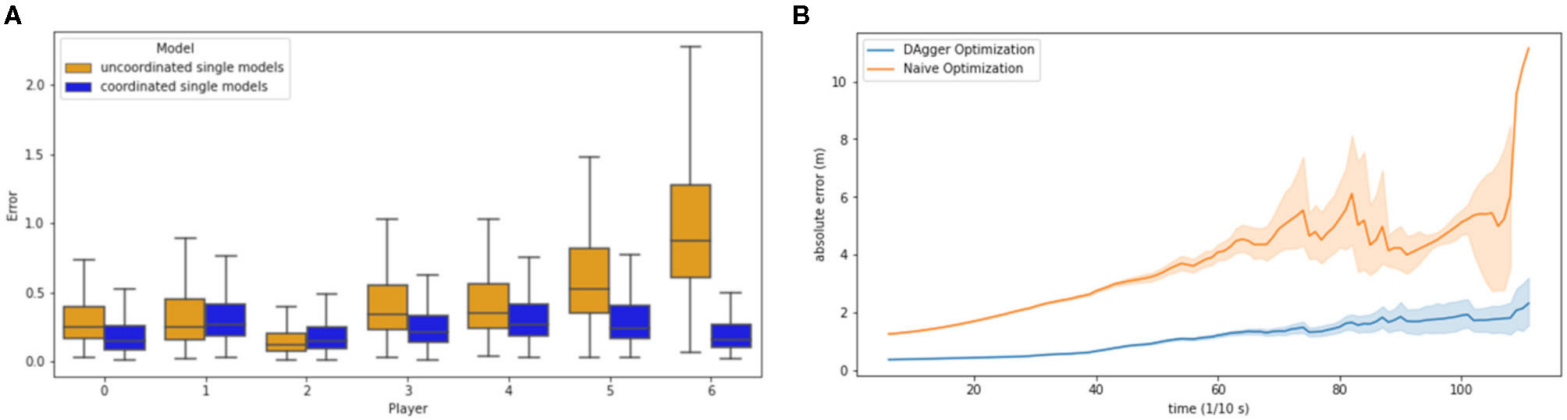
**(A)** Model error for different roles according to specific models. Blue is the coordinated single model. Yellow is the uncoordinated single model. **(B)** Average rolled-out error throughout play, until the quarterback throws a pass. The blue line indicates the rolled-out error under DAgger optimization. The orange line indicates the rolled-out error without DAgger optimization.

In Figure 6A, the impact on a role-based coordination model, in comparison to a static association of roles can be seen. The error for almost every role of the coordinated model is better than the error of the static model. Especially the error of players 5 and 6 are larger than for the coordinated model.

Figure 6B displays the cascading errors (MAE per timestep) occurring due to the rollout of the trajectories. In this study the rollout approach with the described DAgger algorithm to simulate test time is compared to the naive single-agent learning approach. While the naive optimization model error is drifting very strongly with up to 10 m, the DAgger optimization error remains in an acceptable range of about 2 m. DAgger is especially valuable in this approach, as there is no access to an omniscient oracle and, therefore, needs an approximation for deviations in the given trajectories.

**Figure 7:**
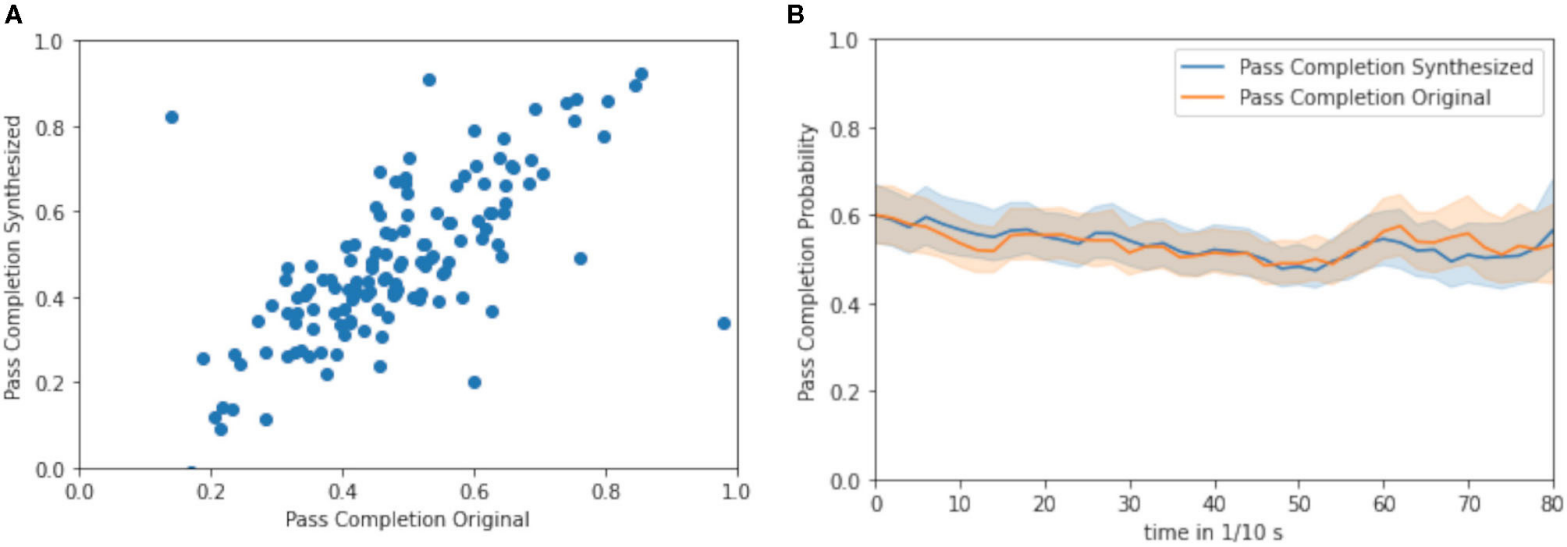
**(A)** Pass completion probability for synthesized trajectories and true trajectories at the time when the quarterback throws the forward pass (R: 0.545) **(B)** Average pass completion over time between synthesized (blue) and true trajectories (orange) of defensive players.

As true running trajectories and simulated trajectories may differ, an impact measurement *via* a “third party,” the trained pass completion probability model is conducted. In Figure 7A, the pass completion probability at the time of the thrown pass in the test set is visible for the respective ghosted trajectories and real trajectories. The *R*-value is 0.545, which indicates the close positive relationship of the pass completion probability of ghosts to the pass completion probability of the observed defenders. Figure 7B displays the average pass completion probability for the synthesized trajectories and the true trajectories throughout the plays in the test set. Although the ghosts run different trajectories during the different plays, in the test set, the average pass completion rate of the ghost is similar to the true pass completion rate, which the ghosts should mimic in the end. With the incorporation of positions of all receivers and defenders, the model is capable of the individual interpretation of the current defensive formation without taking the decision-making of quarterback into account.

## 5. Discussion

### 5.1. Pass Completion Probability

The calculation of the pass completion probability for every player, as proposed by Team (2018), is based primarily on the positioning of players, their closest defenders, or separation from the sideline. Sophisticated models like this are very suitable for analyzing quarterback decision-making and even whole plays in-depth but are too complex to assess team performance. In this approach, the total completion probability is used to compare the ghosting model with the actual running routes. Burke (2019) uses a two-step pass completion probability by first selecting the receiving player and calculating the pass completion probability afterwards. This is a very detailed approach to investigate the decision-making of the quarterback but does not cover defensive team behavior. Hence, the distribution of the targeted player and, therefore, the pass completion probability may change. This approach could be extended by Deshpande and Evans (2020) and the suggested hypothetical pass completion probability, where they investigate if the proper player was chosen for the pass. In the current approach, the decision-making of the quarterback is bypassed, and a pass completion probability by the positions of the receivers and the defenders is computed. This enables the approach to give concise information about the current value of specific opposing positions on the field without biasing the model by a designed combination of pass completion probabilities for every player. Nevertheless, the model accounts for applied pressure on the quarterback by incorporating the kinematic parameters and metadata of defenders, so that indirectly, the model can account for a poorly thrown pass due to the movement of nearby players of the defending team.

### 5.2. Ghosting Model

The objective of the ghosting model is to synthesize realistic defensive behavior. Especially, it should be intrinsically learned to have a team meta behavior, by following a coordinated strategy. In the following section, two examples are investigated and discussed with respect to the evaluation metric.

**Figure 8:**
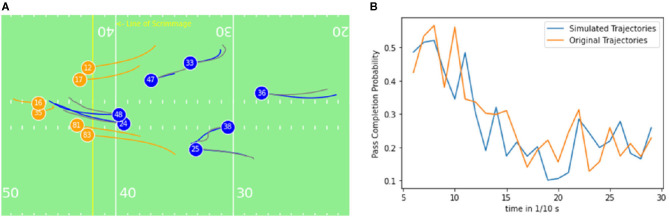
**(A)** Exemplary short play (≈ 3s): The Quarterback (16) throws a short pass to a receiver (12) on the left side of the field. Offensive players are illustrated in blue, defenders orange and ghosted trajectories in gray color. The initial position of the players is represented by the player shirt number. **(B)** Pass completion probability over the course of the play for the ghost/simulated trajectories and true/original movements.

**Figure 9:**
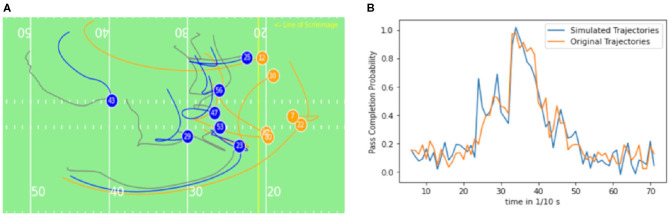
**(A)** Exemplary long play (≈ 7.2 s): The Quarterback (7) throws a pass to a receiver (12) on the right side of the field. Offensive players are illustrated in blue, defenders orange and ghosted trajectories in gray color. **(B)** Pass completion probability over the course of the play for the ghost/simulated trajectories and true/original movements.

Figures 8A, 9A illustrate examples of the observed offensive (blue) and defensive (orange) trajectories for a short and a long play. Parallel to the tracked movement trajectories, the predicted/ghosted movement paths of the defensive players (gray) for the same period are displayed.

In Figure 8A, the true running trajectories are compared with the generated ones, which are interchangeably referred to as ghost trajectories or ghosts in this study. In the figure can be seen, that the ghosts behave similarly to the true players except that the ghosts pressuring the quarterback to decide to run in parallel and the players both tried to tackle the quarterback. The pass was thrown after 3 s. The pass completion probability for the original trajectory and the simulated trajectory, displayed in Figure 8B has the same tendencies, which is closely related to similar positions. Although the model is returning similar tendencies, high noise in the signal relates to a non-perfect pass completion model. Steerability of the ghosting model is included regarding yards to go, the number of downs, and position on the field regarding the distance to the end zone. When changing these variables, no distinguishably different behavior of the ghosted players compared to the initial ghosting outcome can be observed. This indicates a low influence of those variables in the ghosting model for the dataset.

Figure 9A illustrates the behavior of true and ghosted trajectories throughout 7.2 s before the quarterback pass. The ghosted trajectories differ significantly from the actual running trajectories. This is a result of the much larger prediction horizon of the play. The policies/ghosts have significantly more decisions to make and different collaborative behaviors can emerge. After a few seconds, variabilities at the Safety positions (players 43 and 29) can be observed (Figure 9A). The running path of defender number 43 closes some space to the offensive receiver (number 12), the same pattern can be observed for defender number 29. In this study, the ghosts move more backwards and the defender number 29 is closing more to the receiver 12. The collaborating models run a different strategy than the actual players, yet the pass completion probability is similar according to (Figure 9B). As the model takes the defensive behavior of all teams of the NFL into account, the prediction is an average defensive behavior of all teams. Extensive data of specific- teams and players can help to develop team-specific defensive models according to Seidl et al. (2017).

By comparing the coordinated and uncoordinated models in Figure 6A, it can be seen that both safeties have a much larger error than in the coordinated model. This indicates that the safeties are running the most varying strategies and are interchangeable in position (left and right), which yields to the conclusion that the team model cannot capture the strategic changes that are a result of communication between safeties and the reaction to the offensive trajectories. Also, the middle linebacker position (number 4) has a much larger error in the uncoordinated model. These positions seem to have the most different tasks in different strategies, while the cornerbacks (number 1 and 2) and outer linebackers (number 0 and 3) appear to have a more pre determined strategy in the observed formations. Yee et al. (2014) argue that the safety is the most versatile position. Furthermore, Figure 4B displays larger covariances in the hidden Gaussian emissions for the running routes that can be observed for the specific players. Hence, compared to the superior coordinated model, the uncoordinated model helps to understand the influence of global strategy and how it differs across single players.

Respectively, looking into the time evaluation of the pass completion model in Figure 7B, it can be stated that the average pass completion probability is close to the average pass completion probability over the entire period. This indicates that the model is not distinguishing between the timestamp of the trajectory and cannot infer the time when the pass is thrown up to 8 s. Notably, the average pass completion probability of the model over the period for the ghosting model and the original running trajectories is indistinguishable, therefore, the ghosting model infers a similar strategy to the original data and can be used to simulate short and long trajectories before the pass is thrown.

## 6. Conclusion and Future Work

To guarantee the stated accuracy of the predicted positions and make this model helpful for practitioners, the validity of the tracking system needs to be further evaluated. Noteworthy, the sport-specific context turned out to be a challenge for different tracking methods (Hoppe et al., 2018; Linke et al., 2018). Although systems with comparable technology showed promising results in recent validation studies (Blauberger et al., 2021), future research needs to be conducted in the validation of the NFL tracking system.

Deep imitation learning can be mutually adapted to many kinds of team sports with sufficient tracking data at hand. The current study demonstrates that smart feature engineering and reinforcement learning approaches improve the quality of the ghosted trajectories. Investigating a formation with the overall pass completion probability can establish the comparability to run trajectories without comparing the exact position of the players and allowing deviations. However, this lacks the evaluation of the single-player pass completion probability. Upcoming study could include analysis of single player pass completion probability and the variation in those compared to the ghosting models. Furthermore, individualization of players could be included by adding meta-features for every player and, therefore, provide the possibility to compare the performance of individual players in specific plays.

Another drawback of the proposed method is the necessity of a deterministic feature vector. This leads to a massive loss in training data, as it is necessary to determine and adapt to the number of players on the field. In the case, this resulted in a loss of more than 50% of training data for the whole NFL regular season in 2019. With the emergence of graph neural networks and the ongoing research in spatio-temporal graph neural networks (Zhou et al., 2018), this drawback might be resolvable in the near future. Although, the focus of the analysis was kept to pre-throw trajectories for defensive players, the algorithm can be extended to longer trajectories, e.g., movements after the catch. With a sophisticated annotation tool for American football plays, this method could be used to incorporate the versatility of the coverage scheme of defenders. This was not possible with the included data from the NFL dataset 2019 but might be addressed with the work of Dutta et al. (2019). Furthermore, ghosting can be used in the back-end of real-time player sketching. The possible benefit for coaches is also proposed for other sports, like a basketball by Seidl et al. (2017). NFL coaches and analysts can compare their defensive team performance to the league average performance, conduct a hypothetical analysis for specific plays, determine miss behaving defenders, or progress to completely automatic game analysis.

## Data Availability Statement

The original contributions presented in the study are included in the article/supplementary material, further inquiries can be directed to the corresponding author/s.

## Author Contributions

MS designed the experiment and wrote the software. MS and PB wrote the manuscript was written. ML and PB supported the development of the research design, assisted in the interpretation of the data, and reviewed the manuscript. All authors contributed to the article and approved the submitted version.

## Conflict of Interest

The authors declare that the research was conducted in the absence of any commercial or financial relationships that could be construed as a potential conflict of interest.

## Footnotes

^1^ https://www.espn.com/nfl/story/_/id/8379024/nflexplainingexpectedpointsmetric

^2^ https://www.footballoutsiders.com/info/methods#DVOA

^3^ https://www.si.com/nfl/talkoffame/nfl/scoring-defense-the-key-stat-for-super-bowl-contention

^4^ https://en.wikipedia.org/wiki/Imitation
